# LILRB3 (ILT5) is a myeloid cell checkpoint that elicits profound immunomodulation

**DOI:** 10.1172/jci.insight.141593

**Published:** 2020-09-01

**Authors:** Muchaala Yeboah, Charys Papagregoriou, Des C. Jones, H.T. Claude Chan, Guangan Hu, Justine S. McPartlan, Torbjörn Schiött, Ulrika Mattson, C. Ian Mockridge, Ulla-Carin Tornberg, Björn Hambe, Anne Ljungars, Mikael Mattsson, Ivo Tews, Martin J. Glennie, Stephen M. Thirdborough, John Trowsdale, Björn Frendeus, Jianzhu Chen, Mark S. Cragg, Ali Roghanian

**Affiliations:** 1 Antibody & Vaccine Group, Centre for Cancer Immunology, School of Cancer Sciences, Faculty of Medicine, University of Southampton, Southampton General Hospital, Southampton, United Kingdom.; 2 Division of Immunology, Department of Pathology, University of Cambridge, Cambridge, United Kingdom.; 3 Koch Institute for Integrative Cancer Research and Department of Biology, Massachusetts Institute of Technology, Cambridge, Massachusetts, USA.; 4 BioInvent International AB, Lund, Sweden.; 5 Institute for Life Sciences and; 6 Biological Sciences, University of Southampton, Southampton, United Kingdom.

**Keywords:** Immunology, Immunotherapy, Macrophages, Monocytes

## Abstract

Despite advances in identifying the key immunoregulatory roles of many of the human leukocyte immunoglobulin-like receptor (LILR) family members, the function of the inhibitory molecule LILRB3 (ILT5, CD85a, LIR3) remains unclear. Studies indicate a predominant myeloid expression; however, high homology within the LILR family and a relative paucity of reagents have hindered progress toward identifying the function of this receptor. To investigate its function and potential immunomodulatory capacity, a panel of LILRB3-specific monoclonal antibodies (mAbs) was generated. LILRB3-specific mAbs bound to discrete epitopes in Ig-like domain 2 or 4. LILRB3 ligation on primary human monocytes by an agonistic mAb resulted in phenotypic and functional changes, leading to potent inhibition of immune responses in vitro, including significant reduction in T cell proliferation. Importantly, agonizing LILRB3 in humanized mice induced tolerance and permitted efficient engraftment of allogeneic cells. Our findings reveal powerful immunosuppressive functions of LILRB3 and identify it as an important myeloid checkpoint receptor.

## Abstract 2

A panel of LILRB3-specific monoclonal antibodies was generated and used to unravel the immunoregulatory functions of LILRB3 on human myeloid cells using relevant preclinical models.

## Graphical abstract

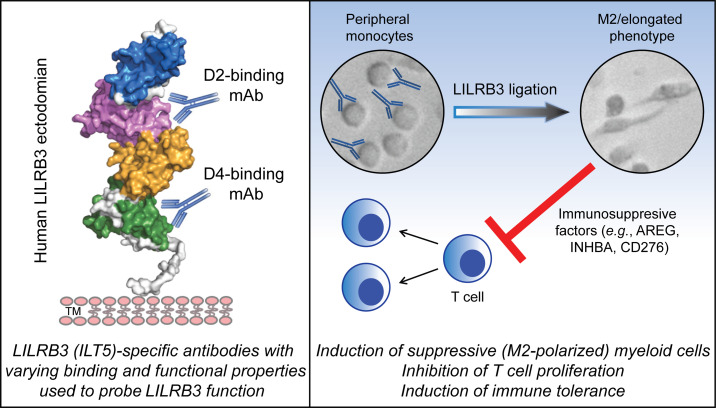

## Introduction

Molecules of the human leukocyte immunoglobulin-like receptor (LILR) family, discovered over 2 decades ago (1, 2), are expressed on leukocytes and are commonly dysregulated in a wide range of pathologies (3–5). There are 5 activating (LILRA1, 2, 4–6), 5 inhibitory (LILRB1–5), and 1 soluble (LILRA3) LILR that together regulate immune responses (3). They display 2, or 4, homologous C-2–type Ig-like extracellular domains but differ in their transmembrane and cytoplasmic regions (2, 6). LILRA have short truncated cytoplasmic tails with charged arginine residues in their transmembrane domains, facilitating association with the immunoreceptor tyrosine-based activation motif–bearing Fcε receptor γ chain to propagate activating signaling cascades (7). Conversely, LILRB have long cytoplasmic tails that contain multiple immunoreceptor tyrosine-based inhibition motifs (ITIMs), which recruit phosphatases such as SHP-1 and SHIP-1 to elicit inhibitory signaling (2, 6). Located at human chromosome 19q13.4, these receptors demonstrate significant allelic variation, with LILRB3, LILRB4 (ILT3), and LILRA6 (ILT8) each displaying at least 15 variants (2, 8–10).

The LILRB molecules are proposed to act as immune checkpoints serving to control and limit overt immune responses (3). In agreement with this, LILRB expression is increased in suppressive (also referred to as alternatively activated or M2) macrophages and tolerogenic dendritic cells (DCs) (11–15). On monocytes, coligation of LILRB1 (ILT2) and LILRB2 (ILT4) with the activatory Fcγ receptor I (CD64) results in SHP-1 activation, decreasing downstream phosphorylation events and intracellular calcium mobilization (16). Engagement of LILRB1 on macrophages by the common HLA-I subunit, β_2_-microglobulin, on malignant cells limits their phagocytic potential (17). Similarly, we and others have shown that ligation of LILRB1, 2, or 4 renders DCs tolerogenic, leading to inhibition of T cell responses (11, 12, 15, 18–21). As such, the engagement of LILRB1 and LILRB2 by their high-affinity ligand HLA-G is an important immunosuppressive pathway at the fetal-maternal interface during pregnancy (22–24) and may be involved in tumor immunoevasion (5).

Although mice do not express LILRs, they possess an orthologous system composed of 2 paired Ig-like receptors (PIRs): the activating PIR-A and the inhibitory PIR-B. PIR-B regulates priming of cytotoxic T lymphocytes by DCs through interaction with MHC class I (MHCI) (25) and negatively influences integrin signaling in neutrophils and macrophages (26). Furthermore, PIR-B regulates the differentiation of myeloid-derived suppressor cells (MDSCs) that aid tumor progression (27).

Among the inhibitory LILRB molecules, LILRB3 (ILT5/LIR3/CD85a), containing 4 extracellular Ig-like domains and 4 cytoplasmic ITIMs, represents an attractive immunomodulatory target because of its relative restriction to, and high expression on, myeloid cells (3, 4). However, due to the lack of specific reagents and model systems, its exact functions and immunoregulatory potential have not been fully explored. In this study, we addressed this by generating a bespoke panel of novel LILRB3-specific mAbs, some of which were used to probe the function of LILRB3 in relevant preclinical platforms. Our data demonstrate that LILRB3 activation confers potent immunoinhibitory functions through reprograming and tolerizing of myeloid cells and suggest that modulating LILRB3 activity may provide exciting new treatment strategies in various disease settings, such as transplantation.

## Results

### Generation and characterization of a panel of fully human LILRB3-specific mAbs.

**Figure 1:**
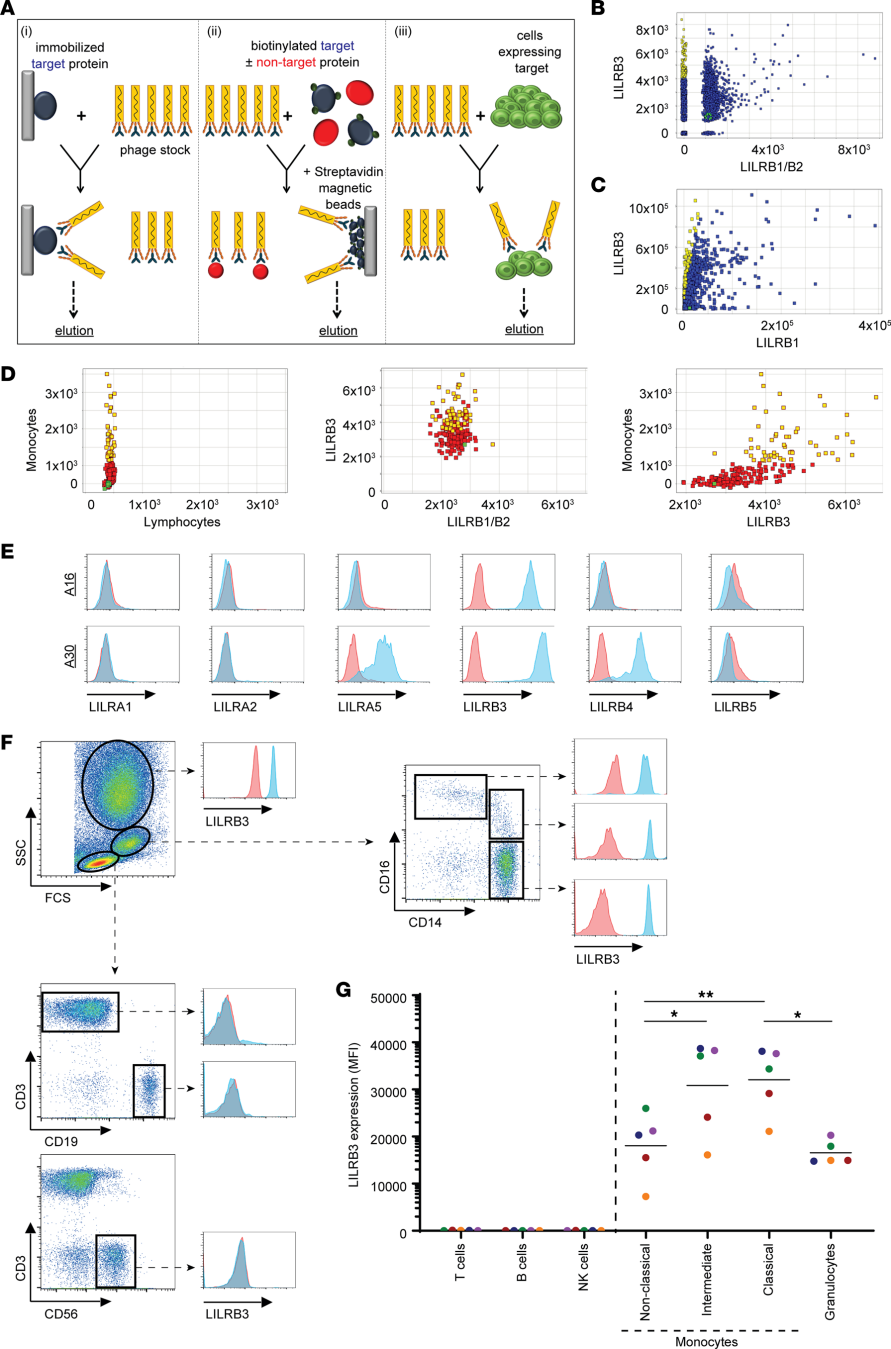
Generation of fully human mAbs against LILRB3. (**A**) Schematic of antibody generation by phage-display via 3 independent “panning” techniques; (i) immobilized target (LILRB3), (ii) biotinylated target and excess nontarget (LILRB1), and (iii) LILRB3-transfected cell lines (from left to right). Biopanning was performed against generated target protein using an scFv library; “nontarget” cross-reactive scFv clones were removed by competition, and target-specific scFv clones were then eluted and converted to a soluble format, sequenced, and screened by various cell- and protein-based assays. (**B** and **C**) Screening of generated LILRB3 clones. (**B**) FMAT and (**C**) ELISA were performed and scFv clones screened against LILRB3 target– and LILRB1/LILRB2 nontarget–transfected CHO-S cells and extracellular LILRB1 protein, respectively. The relative binding to each target was calculated, with target-specific scFv clones depicted in yellow and the irrelevant isotype control shown in green. Nonbinding and cross-reactive scFv clones depicted in blue. (**D**) Screening of LILRB3 scFv clones by high-throughput flow cytometry. PBMCs (left plot) or LILR-transfected CHO-S (middle plot) cells were incubated with His-tagged scFv supernatants, followed by secondary anti-His staining. Where transfected CHO-S cells were used, LILRB1- and LILRB2-transfected cells were used as nontargets for LILRB3. Clones were compared against both gated CD14^+^ monocytes and target-transfected CHO-S cells (right plot). LILRB3-specific clones highlighted in yellow, nonspecific or nonbinding clones in red, and isotype control in green. (**E**) Specificity of LILRB3 clones against human LILR-transfected 2B4 cells. LILRB3 mAbs were tested against cells stably transfected with the indicated LILR family members by flow cytometry; a representative specific clone (A16; top panel) and a nonspecific cross-reactive clone (A30; bottom panel) are shown. (**F**–**G**) Testing the specificity of directly fluorochrome-labeled LILRB3 clones against primary cells by flow cytometry. (**F**) Fresh whole peripheral blood stained with either APC-labeled LILRB3 (represented by clone A16) or an irrelevant human (h) IgG1 isotype control as well as various leukocyte surface markers, as indicated. Dot plots and histograms are representative of multiple donors indicating gating of each leukocyte subset as indicated: T cells, B cells, NK cells, monocytes, and granulocytes. (**G**) Graph showing relative expression of LILRB3 on each leukocyte subset. One-way ANOVA test performed (*P < 0.05; **P < 0.005); *n* = 5 independent donors (each color represents an individual donor). (**E**–**F**) Histogram pink and blue traces indicate staining with irrelevant isotype control or LILRB3 mAb, respectively.

To study the protein expression and function of LILRB3, LILRB3-specific antibodies were identified from a human antibody phage-display library, n-CoDeR (28, 29). Initial alignment analysis of extracellular domains of LILRB1–5 indicated the presence of a limited number of conserved amino acid (a.a.) residues across the LILRB3 ectodomain (Supplemental Figure 1; supplemental material available online with this article; https://doi.org/10.1172/jci.insight.141593DS1), against which specific mAbs could be generated. In this regard, phages binding to the “target” ectodomain of LILRB3 protein (present in solution, coated on a plastic surface, or expressed on cells), and not to the homologous (~65% extracellular homology) “nontarget” LILRB1 ectodomain protein, were selected (Figure 1A and Supplemental Figure 2). To increase specificity and yield, the cross-reactive phages were initially removed through a preselection (negative selection/depletion using “nontarget” proteins), followed by the selection itself (positive selection). Following each selection round, the selected clones were screened against the ectodomains of both LILRB1 and LILRB2 by fluorometric microvolume assay technology (FMAT) and ELISA, and cross-reactive clones were further excluded from the panel. After 3 rounds of phage panning and enrichment, successful selection of clones specific for LILRB3 was reconfirmed by FMAT and ELISA, with target-specific phages converted to soluble single-chain variable fragment (scFv) and screened further (Figure 1, B and C). Successful clones were selected based on binding to LILRB3 and lack of cross-reactivity to LILRB1 and LILRB2. Selected scFv clones (>200) were then sequenced and tested for binding against primary cells and LILRB transfectants using high-throughput flow cytometry (Figure 1D). Subsequently, 46 candidate target-specific clones were converted to human IgG1 (hIgG1) and, in addition to screening against LILRB1–3 transfectants, to exclude those with potential broader LILR cross-reactivity, were screened against a larger panel of LILR-expressing cell lines (Figure 1E). Due to cross-reactivity to one or more other LILR family members, as exemplified by clone A30 (Figure 1E, bottom panel), 30 mAb clones were further excluded at this stage. In total a panel of 16 LILRB3-specific antibodies were identified for further study. These LILRB3-specific clones were further tested and confirmed to have no cross-reactivity to the mouse orthologue, PIR-B (data not shown). A selection of these mAbs were then fluorochrome labeled and used to determine the LILRB3 expression profile on human peripheral blood leukocytes, demonstrating predominant staining of monocytes and to a lesser extent granulocytes (Figure 1, F and G), in agreement with previous reports (2, 3, 6). The immunophenotyping also revealed that LILRB3 expression was significantly higher on circulatory CD14^hi^CD16^–^ classical and CD14^+^CD16^lo^ intermediate monocytes compared with the more inflammatory CD14^+^CD16^hi^ nonclassical monocytes (Figure 1, F and G).

**Figure 2:**
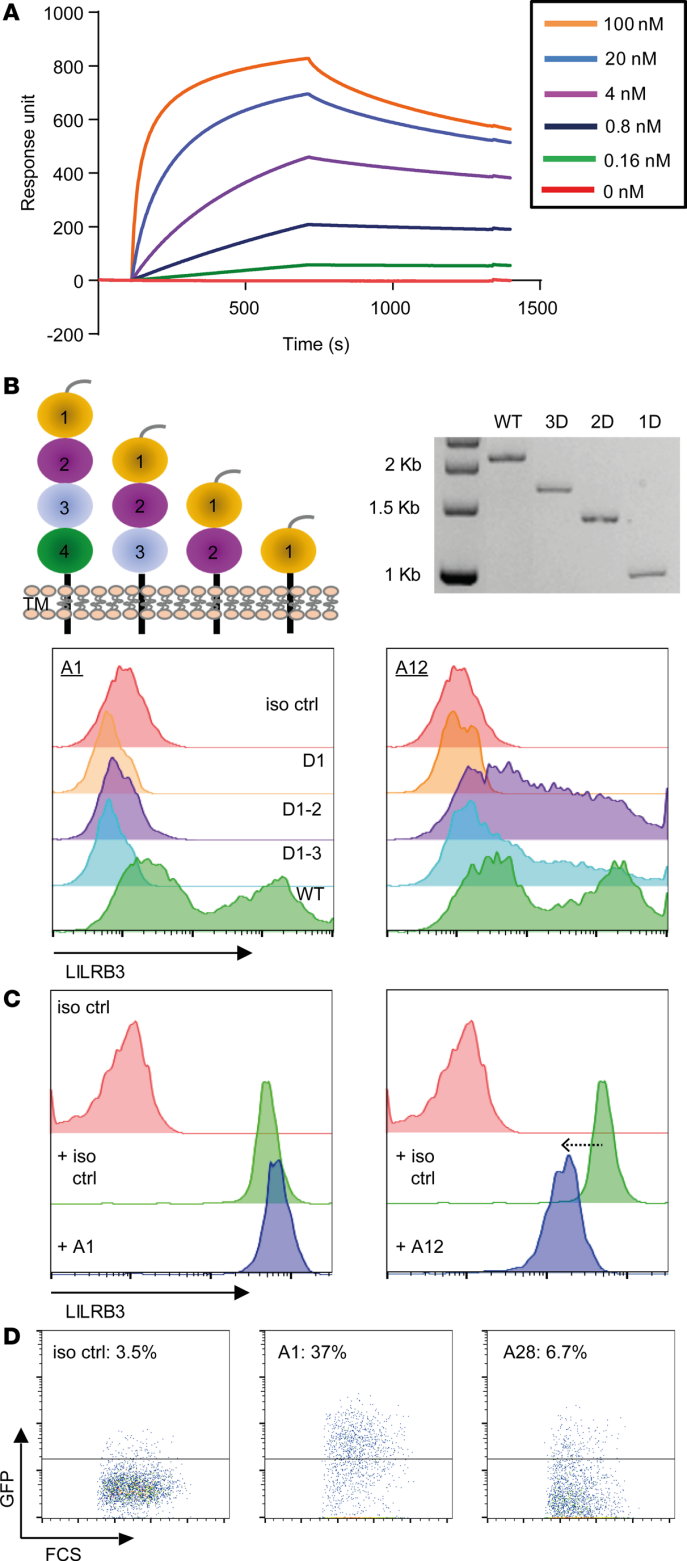
Characterization of LILRB3 antibodies. (**A**) LILRB3 mAb affinity assessed by SPR. LILRB3-hFc recombinant protein was immobilized, and various LILRB3 mAbs flowed across the chip. Representative LILRB3 clone A16 shown. (**B**) LILRB3 domain epitope mapping. HEK293F cells transfected with WT LILRB3 (full-length extracellular portion), D1–3, D1–2, or D1 were stained with LILRB3 clones, followed by an anti-hIgG secondary antibody. Schematic of domain constructs and restriction digest of each DNA construct shown (top panel). Histograms showing staining of 2 representative clones differentially binding to color-coded cells expressing WT (D4), D1–3, D1–2, and D1 (bottom panel; *n* = 3 independent experiments). (**C**) Ability of generated mAbs to cross-block binding of a commercial LILRB3 mAb (clone 222821). PBMCs were stained with unconjugated LILRB3 antibody clones and subsequently stained with a directly conjugated 222821 mAb and analyzed by flow cytometry; representative clones displayed (A1 nonblocking; A12 partial blocking), as indicated. (**D**) LILRB3 2B4 reporter cells were incubated with 10 μg/mL LILRB3 antibodies overnight to assess receptor signaling potential as judged by GFP induction measured by flow cytometry; representative clones with percentage of GFP expression shown (*n* = 2 independent experiments).

The selected LILRB3 mAbs were also tested for their specific binding properties. Surface plasmon resonance (SPR) analysis showed that all LILRB3-specific clones bound to recombinant LILRB3-hFc protein in a dose-dependent manner (as represented by A16; Figure 2A) and displayed a range of affinities (Table 1). Interestingly, all mAbs had similar association rates (~10^5^) but varied in their dissociation rates by 3 orders of magnitude (~10^–3^ to 10^–6^).

Cell surface epitope-mapping studies were then performed and compared with a commercial mAb (clone 222821), using a series of LILRB3 extracellular domain (D) mutants displaying either all 4 Ig domains (WT), or 3 domains, 2 domains, or 1 domain, transiently transfected into HEK293F cells. Two distinct groups of mAbs were identified: those that bound to the WT, D3-expressing, and D2-expressing cells (including clone 222821 and exemplified by A12) and those that bound only to the WT-transfected cells (exemplified by A1) (Figure 2B). Although conserved a.a. residues were present throughout the ectodomain (Supplemental Figure 1), the selected mAbs were shown to bind within either D2 or D4 (6/16 and 10/16 clones, respectively; Table 1), perhaps indicating improved accessibility for these regions within the 3D structure. In agreement with this, subsequent blocking assays confirmed that a number of D2-binding mAbs reduced the binding of the commercial mAbs (e.g., A12), suggesting shared or related epitopes, while others did not (e.g., A1), confirming binding to discrete epitopes (Figure 2C and Table 1).

Subsequently, reporter cells transfected with a chimeric receptor expressing the extracellular domain of LILRB3, fused with the human CD3ζ cytoplasmic domain, were used to investigate whether the generated mAbs were able to cross-link the receptor. Cross-linking results in the production of nuclear factor of activated T cells activation and the subsequent expression of GFP and is indicative of agonistic potential (30). Using these cells, we were able to identify 2 distinct groups of LILRB3 mAbs, those with “agonistic” activity capable of inducing signaling upon binding to the receptor (e.g., A1) and those that were inert (e.g., A28) (Figure 2D). Collectively, these data demonstrate that highly specific, fully hIgG1 mAbs were raised against LILRB3, amenable to the comprehensive evaluation of LILRB3 function.

### LILRB3 ligation modulates T cell activation and proliferation.

**Figure 3:**
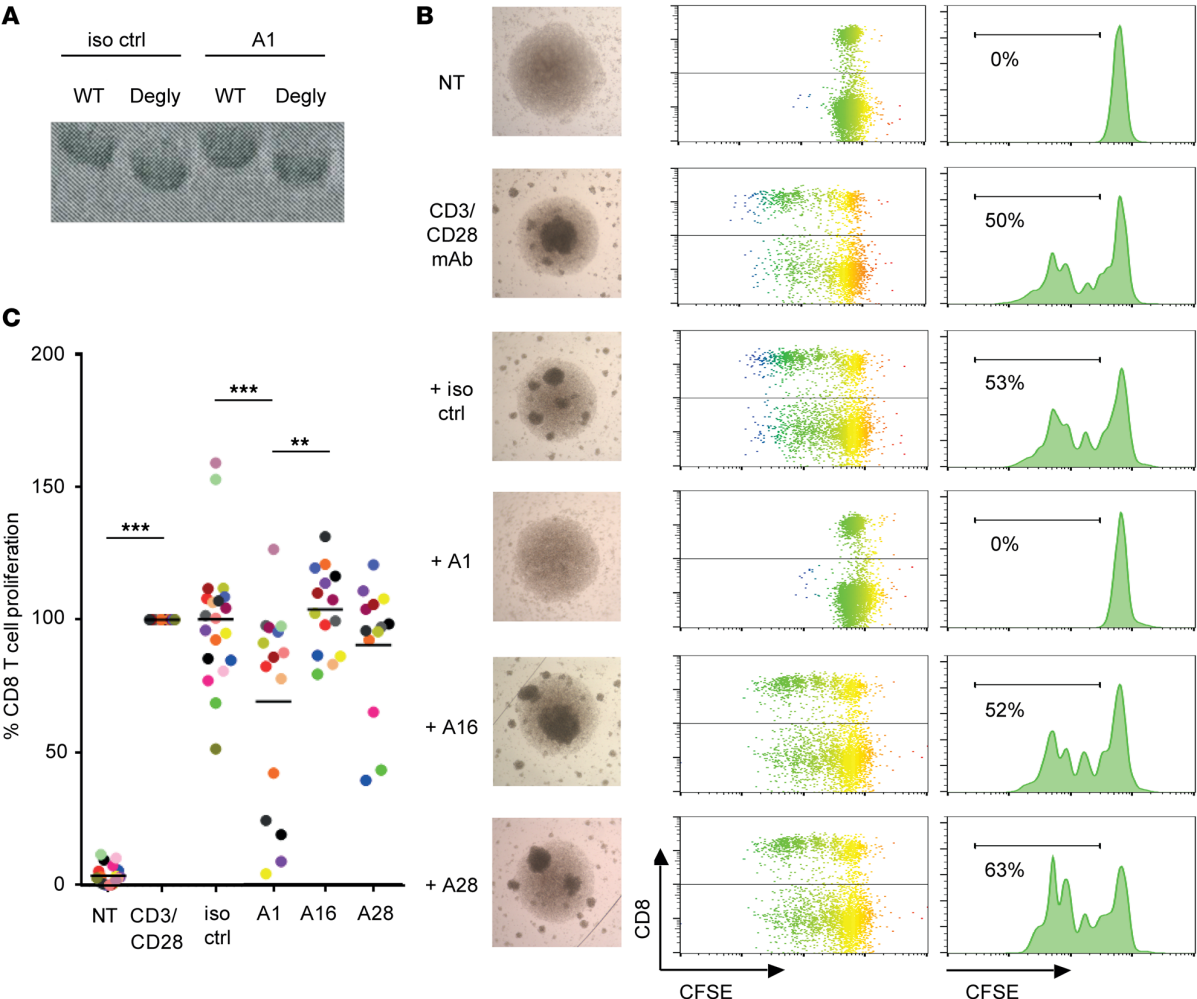
LILRB3 ligation regulates T cell activation and proliferation. CFSE-labeled PBMCs were stimulated with antibodies against human CD3 (0.02 μg/mL) and CD28 (5 μg/mL) in the presence or absence of isotype control (iso ctrl) or LILRB3 mAb (10 μg/mL) and proliferation measured through CFSE dilution after 3–5 days. (**A**) LILRB3 mAbs were deglycosylated (Degly) through PNGase treatment, as confirmed by SDS-PAGE; representative clone A1 shown. (**B**) Assessing T cell activation and proliferation following treatment. Light microscopy images following PBMC stimulation in culture. CD8^+^ T cell proliferation was assessed through CFSE dilution; plots and images from a donor with profound A1-induced inhibition shown, histograms (% proliferation indicated) and microscopy images shown (original magnification, ×10). (**C**) Assessing the effects of deglycosylated LILRB3 mAbs on T cell proliferation. CFSE dilution of CD8^+^ T cells, treated with the representative LILRB3 mAb, was assessed by flow cytometry. Data normalized to anti-CD3/CD28–treated samples and mean represented by solid bars. One-way ANOVA performed (**P < 0.005; ***P < 0.0005); *n* = 13–20 independent donors (each color represents an individual donor).

Accordingly, using a select number of mAbs, we sought to investigate the immunomodulating effect of the LILRB3 mAbs on cellular effector functions. LILRB1 has previously been shown to directly inhibit T cell responses by causing dephosphorylation of the CD3 signaling cascade, and, in addition, has the potential to negatively regulate T cell activation by competing with CD8 for HLA-I binding (31, 32). Moreover, LILRBs can indirectly inhibit T cell responses by rendering antigen-presenting cells (APCs), such as monocytes and DCs, tolerogenic (14, 18, 33). To investigate the immunomodulatory potential of LILRB3 and its ability to regulate adaptive immune responses, we used a T cell proliferation assay incorporating fresh PBMCs isolated from healthy human donors, as before (34). Fcγ receptors (FcγRs) help mediate the effects of hIgG (35). Therefore, to study the direct F(ab′):LILRB3-mediated effects of the mAb on T cell proliferation, they were first deglycosylated to reduce FcγR-IgG interactions. SDS-PAGE showed a decrease in molecular weight of deglycosylated mAbs compared with WT controls, indicative of successful deglycosylation (Figure 3A). The mAbs were then introduced to a T cell proliferation assay where CD3 and CD28 antibodies elicited cell clustering and CFSE dilution, indicative of a significant increase in CD8^+^ T cell proliferation, compared with nontreated controls (Figure 3, B and C). Clone A1, shown to be an agonist (Figure 2D), significantly inhibited CD8^+^ T cell proliferation in this assay when compared with the isotype control (Figure 3, B and C). Similarly, the commercial antibody (clone 222821) substantially inhibited T cell proliferation (data not shown). Other LILRB3-specific mAbs had either no or subtle effects, as represented by clones A16 and A28. These data demonstrate that LILRB3 ligation by agonistic mAbs suppresses T cell responses, whereas other clones confer no inhibitory effects. Similar effects were also observed when considering CD3^+^CD8^–^ T cells (predominantly CD3^+^CD4^+^ T cells; Figure 3C and not shown). There was notable donor variation in the T cell proliferation assay, which is partly due to the nature of this assay and may also reflect differing expression levels and/or LILRB3 polymorphic variants present on the myeloid cells — none of which were formally tested here. When the assay was repeated with isolated T cells, no inhibition was seen, confirming that APCs within the PBMC mixture, most likely monocytes, were responsible for the effects observed (Supplemental Figure 3), as expected, given the lack of expression of LILRB3 on T cells (Figure 1, F and G).

### LILRB3 ligation induces immune tolerance in humanized mice.

**Figure 4:**
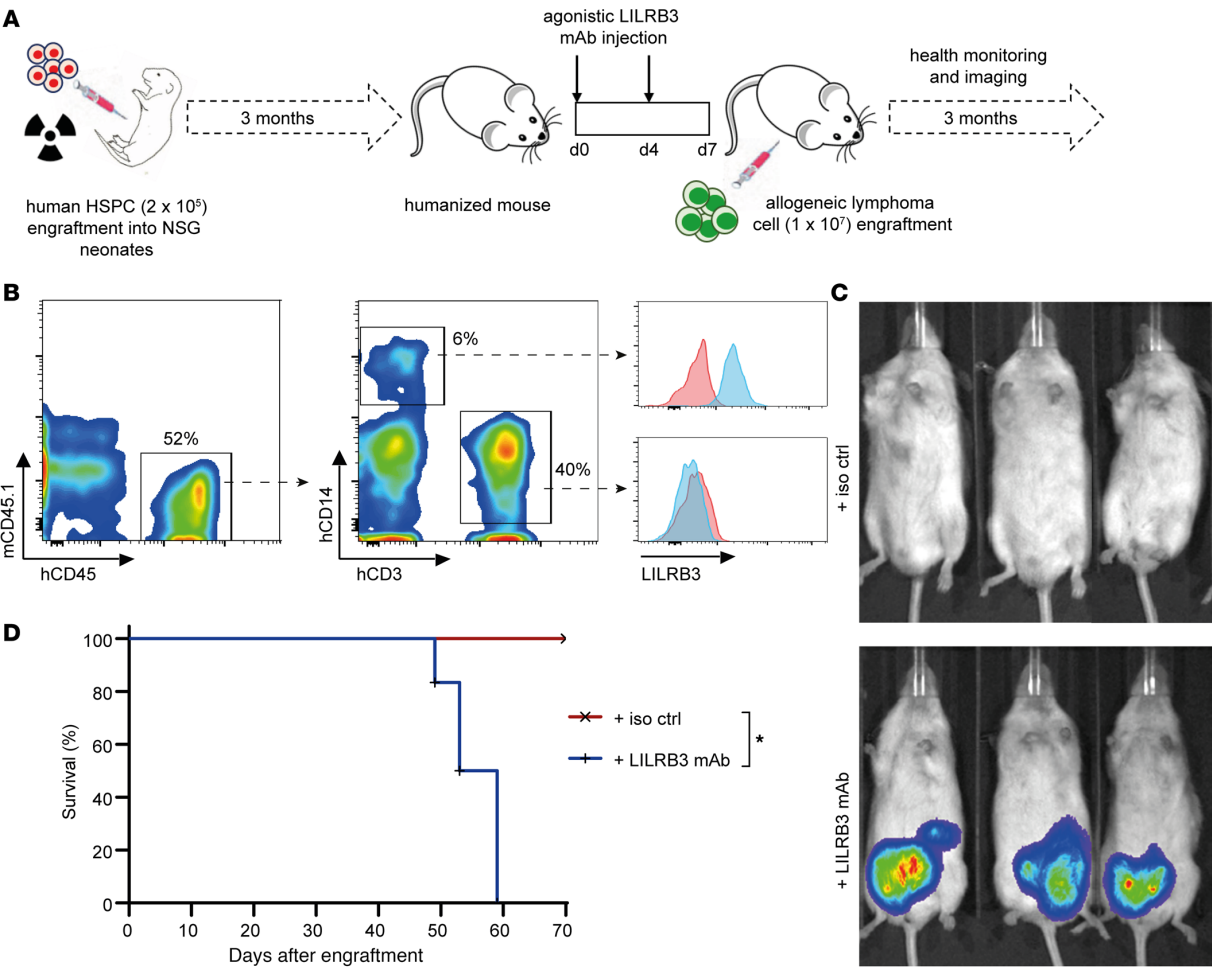
LILRB3 ligation induces tolerance in vivo. (**A**) Schematic of the generation of humanized mice and subsequent treatment regimens and monitoring. (**B**) Expression of LILRB3 on human myeloid cells in humanized mice. Representative flow cytometry plots (gated on live single cells) showing gating strategy and the restricted expression of LILRB3 on hCD45^+^ peripheral blood hCD14^+^ myeloid cells; isotype control in pink and LILRB3 mAb staining depicted in blue. (**C**) The effect of agonistic LILRB3 mAb on engraftment of allogeneic cells in humanized mice. Age- and sex-matched humanized mice were injected with 200 μg LILRB3 mAb (clone A1) or an isotype-matched (hIgG1) control mAb (iso ctrl) on day 0 and 4, i.v. and i.p., respectively. On day 7, mice were injected i.p. with 1 × 10^7^ nonautologous luciferase^+^ human lymphoma cells. Lymphoma cell growth was monitored over time using an IVIS imager, and (**D**) humanized mice were sacrificed upon the development of signs of terminal tumor development. Survival data were analyzed using log-rank test (*P < 0.01); representative data from 3 independent experiments (3 individual HSPC donors) shown (*n* = 3 mice/group).

Given these data showing that T cells could be suppressed following LILRB3 ligation on myeloid cells, we next investigated the possible effects of LILRB3 modulation in an allogeneic engraftment model using humanized mice, previously reconstituted with primary human fetal hematopoietic stem/progenitor cells (HSPCs) (Figure 4A). Characterization of peripheral blood leukocytes and bone marrow of adult humanized mice demonstrated that LILRB3 was expressed on, and restricted to, myeloid cells, but not lymphocytes, similar to humans (Figure 4B and Supplemental Figure 4). We recently showed that allogeneic human lymphoma cells are readily rejected in humanized mice due to HLA mismatch (36). To test the potential of LILRB3 ligation to suppress the allogeneic immune response, adult humanized mice were treated with the agonistic LILRB3 mAb (A1) and the engraftment of allogeneic human B cell lymphoma cells, derived from an unrelated donor (36, 37), was monitored over time (Figure 4A). LILRB3 mAb treatment was able to induce a state of tolerance in vivo and led to a successful engraftment of the donor allogeneic cells (Figure 4C). Accordingly, LILRB3-treated tumor-bearing humanized mice subsequently succumbed to disease with high tumor burden, whereas isotype control–treated mice readily rejected the lymphoma cells without morbidity (Figure 4D). These observations corroborate our in vitro functional assays and identify LILRB3 as a key regulator of immune tolerance in an allotransplant setting. Given the expression pattern of LILRB3 on myeloid but not lymphocytic cells in both the human PBMCs and humanized mice, we sought to explore the effects of the LILRB3 mAbs on these cells.

### LILRB3 ligation leads to transcriptional modification and M2 skewing of human APCs.

**Figure 5:**
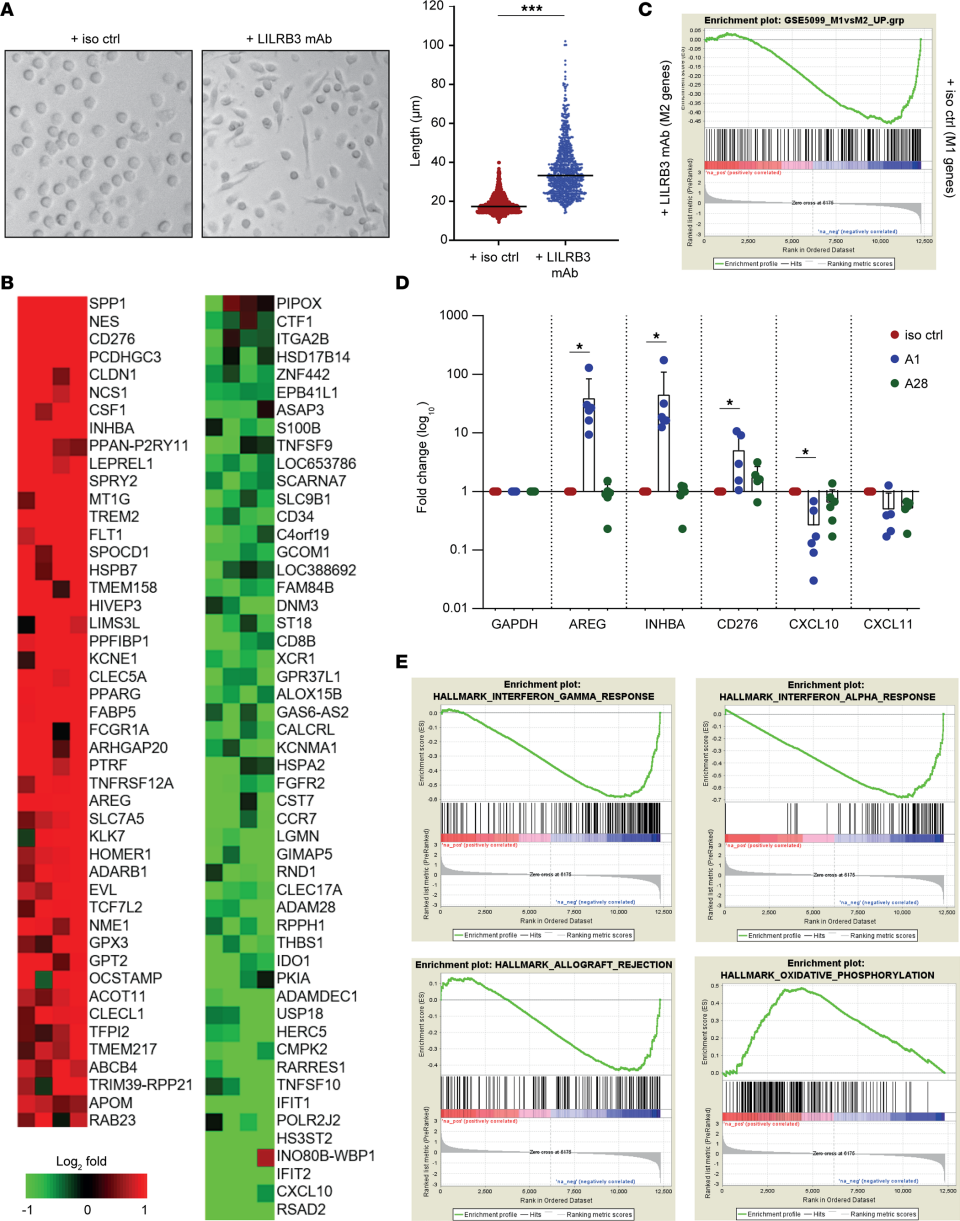
Human LILRB3 ligation reprograms human primary myeloid cells. Freshly isolated human peripheral CD14^+^ monocytes were treated with an isotype control (iso ctrl) or a human LILRB3 mAb (clone A1) and then assessed. (**A**) Agonistic LILRB3 mAb (clone A1) affects monocyte morphology. Light microscopy images following overnight treatment of freshly isolated CD14^+^ monocytes with indicated mAbs in culture (original magnification, ×10; left panel). Images of treated monocytes were analyzed and length of monocytes quantified (right panel). A total of 200–500 individual cells were analyzed per image. Combined data from 3 independent donors shown; lines indicate median; 2-tailed paired *t* test performed (***P < 0.0001). (**B**) Transcriptomic analysis of LILRB3-treated monocytes reveals upregulation of M2-associated genes compared with controls. RNA was extracted from cells following mAb treatment (~18 hours) and subjected to RNA-Seq. Red depicts genes that were significantly upregulated, and green depicts genes that were significantly downregulated compared with isotype control–treated cells (*n* = 5–6 independent donors). (**C**) Ligation of LILRB3 on primary human CD14^+^ monocytes downregulated M1-associated genes. GSEA graph showing a significant enrichment for M1-polarizing genes in LILRB3-treated monocytes versus isotype control, respectively. UP; upregulated, normalized enrichment score (NES) = –1.68; family-wise error rate (FWER); *P* < 0.001. (**D**) qPCR analysis of selected genes following LILRB3 ligation on monocytes using an agonistic LILRB3 mAb (A1), a nonagonistic LILRB3 mAb (A28), or an isotype control (iso ctrl). Data were normalized to GAPDH mRNA levels and standardized to the levels of isotype control–treated monocytes. Fold difference data were log_10_ transformed. One-way ANOVA with Bonferroni’s multiple-comparisons test was performed (*P < 0.005). (**E**) GSEA showing negative correlation with IFN-γ (NES = –2.17; FWER *P* < 0.001), IFN-α (NES = –2.3; FWER *P* < 0.001), and allograft rejection (NES = –1.58; FWER *P* = 0.14) signaling elements and positive correlation with oxidative phosphorylation (NES = 2; FWER *P* < 0.001).

To investigate the pathways and factors involved in LILRB3-mediated immunosuppression, we next investigated the transcriptomic changes in monocytes following LILRB3 engagement. Short-term (~18-hour) in vitro treatment of freshly isolated human peripheral CD14^+^ monocytes with the agonistic LILRB3 mAb (A1) caused a dramatic shift in their phenotype (Figure 5A), with the cells displaying a significantly more elongated morphology (*P* < 0.0001) resembling immunosuppressed M2 macrophages (38). In accordance with this, RNA-Seq analysis revealed that ligation of LILRB3 on monocytes induced a signature resembling “M2-skewed” immunosuppressive macrophages (Figure 5B). Concurrently, the expression of genes associated with “M1-skewed” immunostimulatory macrophages was downregulated in LILRB3-ligated monocytes (Figure 5, B and C). These data were confirmed by quantitative PCR (qPCR) for a number of the differentially regulated genes on a further 6 donors (Figure 5D). As further evidence, we showed that the effects were dependent upon LILRB3 agonism because treatment of monocytes with a nonagonistic LILRB3 mAb (A28), despite binding the same domain, did not affect monocyte phenotype or gene expression (Figure 5D). Gene set enrichment analysis (GSEA) of the RNA-Seq data showed a positive correlation with gene signatures reported for suppressive macrophages, e.g., oxidative phosphorylation (39). Conversely, LILRB3-ligated monocyte gene signatures negatively correlated with those reported for inflammatory macrophages, e.g., IFN-γ and IFN-α responsive elements, as well as allograft rejection (Figure 5E), in line with our in vivo observations (Figure 4). In summary, these data show that LILRB3 activation results in significant phenotypic and transcriptional alterations in human primary myeloid cells, leading to potent inhibition of downstream immune responses.

## Discussion

We previously demonstrated that ligation of LILRB1 on human DCs induces a tolerogenic phenotype, hindering T cell responses (18, 40). In this study, we investigated another inhibitory LILR family member, LILRB3, whose function, largely due to lack of suitable reagents and experimental systems, is not yet fully determined. Limited previous studies investigated the consequences of LILRB3 ligation on granulocytes and have demonstrated its inhibitory function on neutrophils (41) and basophils (42) in culture. Here, we largely concentrated on myelomonocytic cells and the subsequent regulation of adaptive immune responses. We, therefore, initially generated and characterized an extensive panel of fully human mAbs with specificity for LILRB3 through a number of stringent panning and selection processes. Those clones showing cross-reactivity to other human LILR family members were excluded. Immunoprofiling of circulatory leukocytes from healthy donors using these highly specific mAbs confirmed the reported expression of LILRB3 on myelomonocytic and granulocytic cells, but not on lymphocytes (2, 3, 6). This pattern of expression on myeloid and granulocytic but not lymphoid cells was confirmed in a large cohort of independent donors (>50), suggesting that, despite the polymorphic nature of LILRB3 (2, 9, 43), the selected antibodies recognize many, if not all, variants, which is important for the development of these reagents for therapeutic applications. Subsequent analysis showed that the LILRB3 mAbs displayed a range of affinities, albeit all within the nanomolar (nM) range, with similar on rates, but off rates differing over 3 orders of magnitude. *K_D_* values in the low nanomolar range are generally considered viable drug candidates; rituximab, for example, has an 8 nM affinity for its target, CD20 (44). This suggests that the LILRB3 mAbs generated here have potential as therapeutic agents. However, because LILRB3 shares high sequence homology (>95%) in its extracellular domain with LILRA6, there is a possibility that our LILRB3 mAbs may also recognize shared epitopes on LILRA6, if coexpressed (9). These initial data might receive further evidence from other reagents as well as investigation as to whether LILRA6 protein is detectable in leukocyte subsets, e.g., using proteomics approaches similar to a recent study with neutrophils (41). Epitope-mapping experiments revealed that the specific LILRB3 mAbs reported herein were generated against 2 specific ectodomains, either Ig-like domain 2 or 4. Interestingly, none of the generated specific LILRB3 mAbs bound to Ig-like domain 1 or 3, suggesting that these domains may not contain epitopes that are unique for LILRB3, or more likely those unique residues are not exposed/accessible. Collectively, these data confirm that our LILRB3 mAbs will be useful tools for dissecting LILRB3’s molecular mechanisms and may additionally have therapeutic benefits in relevant pathologies.

The ability of the LILRB3 mAbs to influence T cell responses was variable, with some inhibiting proliferation, while others resulted in modest increases in proliferation, supportive of agonistic or blocking properties, respectively. Similar to LILRB1 (17, 18, 21), these effects are likely through manipulations of APCs, specifically monocytes, because they are the only cells expressing LILRB3 in the culture. In support of this, the agonistic LILRB3 mAbs did not suppress T cell proliferation in the absence of monocytes. Binding epitopes influence the ability of mAbs to modulate receptor function in many systems (35, 45), and so it was unsurprising to see LILRB3 mAbs capable of differing functions. However, the D4-binding A1 mAb was a strong inhibitor of proliferation, whereas other D4-binding mAbs (e.g., A28) had no significant effect. Therefore, domain-specific epitopes did not seem to correlate directly with LILRB3 mAb–mediated effector cell functions and may not be predictive of LILRB3 mAb function per se. Further detailed analyses, e.g., surface alanine scanning mutagenesis (45) and/or structural studies, are required to define the specific extracellular epitopes engaged by the selected LILRB3 mAbs and to investigate their influence on receptor activity.

Our observations demonstrating immunoinhibitory activities downstream of LILRB3 were further confirmed in the reconstituted humanized mouse model. In this system, where LILRB3 is present only on the hematopoietic cells, and predominantly monocytes, in the absence of appreciable numbers of neutrophils, ligation of LILRB3 with an agonistic LILRB3 mAb before injection of allogeneic lymphoma cells (36, 37) induced tolerance in vivo and enabled subsequent tumor cell engraftment. This demonstrates the capacity of LILRB3 to exert profound immunosuppressive effects that may be exploited in therapeutic settings, such as autoimmunity and transplantation, where transient induction of immune tolerance will be beneficial. Similar observations were previously reported using a LILRB1 transgenic mouse model, where interactions between LILRB1 and MHCI or HLA-G expanded MDSCs and prolonged allogenic graft survival in vivo (46, 47).

Although typically regarded as an orphan receptor, earlier studies suggest that LILRB3 may associate with cytokeratin-associated proteins such as those exposed on necrotic cancer cells (30). Others have also identified angiopoietin-like protein 5 and bacteria, such as *Staphylococcus aureus*, as a source of potential ligands (48, 49). Therefore, our data provide a strong mechanism of action whereby such endogenous or pathogenic ligands may be able to subvert immune responses by ligating LILRB3 during an ongoing immune response.

To investigate the pathways and factors involved in LILRB3-mediated immunosuppression, we investigated the transcriptomic changes in isolated peripheral myeloid cells following LILRB3 ligation. Over 100 genes were differentially regulated in primary human monocytes following LILRB3 ligation, some of which are known to be associated with M2-polarized macrophages (13, 50). Amphiregulin (*AREG*) was among the genes whose expression was markedly upregulated in LILRB3-ligated monocytes. AREG is an epidermal growth factor–like growth factor, responsible for inducing tolerance and immunosuppression, via various mechanisms, including enhancement of Treg activity (51). Furthermore, AREG is overexpressed in tumor-associated DCs (52) and suppressive/M2 macrophages (53) and has been suggested to play a crucial role in immunosuppression and cancer progression (54). Similarly a number of other candidates, e.g., *activin A* (55) and *CD276* (56), known for their immunosuppressive functions in myeloid cells were induced upon LILRB3 ligation. Although not formally tested, LILRB3-induced production of soluble factors, such as AREG, by myeloid cells may promote the expansion and suppressive capacity of Tregs in the PBMC cultures. Similarly, the reported upregulation of immunoinhibitory receptors, such as CD276 (B7-H3), upon LILRB3 ligation on myeloid cells may restrain the activation of T cells and their proliferative capacity (56, 57). In addition, GSEA demonstrated that ligation of LILRB3 on monocytes substantially affected a number of key pathways and functions. Interestingly, LILRB3-ligated monocytes had a gene signature associated with oxidative phosphorylation, which is the metabolic pathway adapted by M2-polarized myeloid cells and is important for their immunosuppressive activities (39). Such LILRB3-inducible immunosuppressive receptors or soluble factors may be responsible for the suppression observed in our T cell assays and for the induction of tolerance in the humanized mouse model. Our ongoing efforts aim to interrogate these findings further and define the mechanisms responsible for LILRB3-mediated suppression of immune responses at molecular and cellular levels, e.g., via siRNA knockdown or neutralization of AREG in monocyte cultures and validation of differentially regulated genes in the humanized mouse models. A recent study investigating the mode of action of glatiramer acetate (Copaxone), a peptide-based drug licensed in the late 1990s, used to treat patients with the relapsing-remitting form of multiple sclerosis that ameliorates autoimmunity, identified LILRB2 and LILRB3 as potential ligands (58). On the other hand, blocking human LILRB2 with antagonistic mAbs on human myeloid cells is able to promote their proinflammatory activity and enhance antitumor responses in preclinical models (58); a LILRB2 mAb (MK-4830) recently entered phase I clinical trials (ClinicalTrials.gov NCT03564691) for advanced solid tumors. Furthermore, recent data by Zhang and colleagues suggest that LILRB4 signaling in leukemia cells mediates T cell suppression and supports tumor cell dissemination to distal organs (59). These recent compelling reports further support our findings, demonstrating that ligation of human LILRB3 induces immunosuppression via reprogramming of myeloid cells (i.e., reducing M1-like maturation and promoting suppressive function).

In conclusion, we generated a specific panel of human LILRB3 mAbs, binding to unique residues located within ectodomain 2 or 4, and used the strongest agonistic clone (A1) to reveal LILRB3’s potent immunoregulatory functions on myeloid cells using bespoke preclinical models. Our data demonstrated that LILRB3 engagement on primary human myeloid cells exerts potent immunoinhibitory functions and that LILRB3-specific mAbs are potentially powerful immunomodulatory agents, with broad applications ranging from transplantation to autoimmunity and beyond, where fine-tuning of immune responses through myeloid cell activity is desired.

## Methods

### Cell culture

Cell lines were grown at 37°C in RPMI 1640 medium supplemented with 10% heat-inactivated fetal calf serum (FCS) (MilliporeSigma), 100 U/mL penicillin-streptomycin, 2 mM glutamine, and 1 mM pyruvate (Thermo Fisher Scientific) in a humidified incubator with 5% CO_2_, FreeStyle 293F medium, in 8% CO_2_, shaken at 130 rpm, or FreeStyle CHO medium (Thermo Fisher Scientific) with 8 mM glutamine, in 8% CO_2_, shaken at 140 rpm.

### Antibody generation and production

#### Generation of LILRB3 antibodies.

Generation of LILRB3-specific mAbs was performed using the n-CoDeR phage-display library (28). Three consecutive panning rounds were performed, as well as a prepanning step. In the panning, human (h) Fc fusion proteins containing the extracellular domains of LILRB1 and LILRB3 (LILRB-hFc) were used as “nontarget” or “target,” respectively. These proteins were produced in transiently transfected HEK293F cells (ATCC) followed by purification on protein A, as described previously (35). CHO-S cells (ATCC) transiently transfected to express the various LILRB proteins were also used as targets/nontargets in the panning.

In panning 1, BioInvent n-CoDeR scFv were selected using biotinylated in-house–produced recombinant LILRB3-hFc fusion proteins (captured with streptavidin-coated Dynabeads, Thermo Fisher Scientific) with or without competition or LILRB1-hFc coated onto etched polystyrene balls (Polysciences) or plastic immunotubes. Binding phages were eluted by trypsin digestion and amplified on plates using standard procedures (60). The amplified phages from panning 1 were used for panning 2, the process repeated, and the amplified phages from panning 2 used in panning 3. In the third panning round, however, amplified phages from all 3 strategies were combined and selected against LILRB-expressing CHO-S cells, before making the final LILRB3-specific mAb selection.

Next, the LILRB3-positive scFv from the enriched phage repertoires from panning 3 were subcloned to allow soluble scFv expression in *E*. *coli*. The soluble scFvs expressed by individual clones were tested for binding against LILRB-transfected CHO-S cells using FMAT and recombinant LILRB protein by ELISA. This allowed the identification of clones binding specifically to LILRB3. Clones were then further reduced in a tertiary screen against CHO-S cells expressing LILRB1–3 and primary cells (PBMCs) using a high-throughput flow cytometry screening system, with data analyzed by TIBCO Spotfire software (TIBColorado). Clones showing specific patterns of binding to LILRB3 were sequenced, yielding LILRB3-specific mAbs.

#### Production of full-length IgG.

The unique scFv identified above were cloned into a eukaryotic expression system allowing transient expression of full-length IgG in HEK293-EBNA cells. The antibodies were then purified from the culture supernatants using Protein A affinity chromatography as previously described (35).

#### Production of deglycosylated IgG.

To allow dissection of Fc- and F(ab′)-dependent effector functions, IgGs were deglycosylated using PNGase F (Promega) with 0.05 U PNGase/μg IgG, at 37°C for at least 15 hours. Deglycosylation was confirmed by reduction in size of the heavy chain on SDS-PAGE.

### Production of domain mutant constructs

Using WT LILRB3 cDNA isolated from healthy donor PBMCs, a series of domain-mutant DNA constructs were generated by overlap PCR to express 1, 2, or 3 LILRB3 Ig-like domains (with domains identified based on annotations in UniProt) for comparison to WT LILRB3 (4 domains). The gene constructs were then cloned into pcDNA3.

### Cell transfections

HEK293F cells (1 × 10^7^) were transiently transfected with 10 μg of plasmid DNA by lipofection using 233 fectin with Opti-Mem 1 Media (Thermo Fisher Scientific).

### Preparation of human leukocytes

PBMCs were isolated from leukocyte blood cones (Blood Transfusion Services, Southampton General Hospital) by gradient density centrifugation using lymphoprep (Axis-Shield) and used for subsequent experiments, as before (61).

### Flow cytometry

For cell surface staining, human PBMCs, whole blood, or leukocytes from humanized mice were blocked with 10% human AB serum (MilliporeSigma) for 10 minutes on ice and then stained with the relevant APC-labeled LILRB3 mAb or hIgG1 isotype (BioInvent), alongside the following cell surface markers: human CD14-PE (clone 61D3; eBioscience, Thermo Fisher Scientific), CD20-A488 (rituximab; in-house), CD3-PE-Cy7 (clone HIT3a), CD56-APC-Cy7 (clone 5.1H11) or CD15-Pacific Blue (clone HI98), CD15-PE and CD66B-FITC (clone G10F5), CD45-APC-Cy7 (clone 2D1), and mouse CD45.1-PerCP (clone A20) (all BioLegend). Cells were stained for 30 minutes at 4°C and then washed twice, first in 10% red blood cell (RBC) lysis buffer (Serotec) for PBMCs or 1× Erythrolyse RBC Lysing Buffer (Bio-Rad) for whole blood, then with FACS wash (PBS, 1% BSA, 10 mM NaN_3_), before acquisition on a FACSCalibur or FACSCanto II (BD Biosciences) and analysis with FlowJo software (BD Biosciences).

For assays to determine if mAbs bound to similar cross-blocking epitopes, 1 × 10^6^ PBMCs were blocked with 2% human AB serum for 10 minutes and stained with 10 μg/mL unconjugated LILRB3 mAbs for 30 minutes at 4°C. The cells were then stained with directly conjugated LILRB3 mAbs (clone 222821; mouse IgG2a; R&D Systems, Bio-Techne) for 20 minutes at 4°C, before washing and acquisition using a FACSCalibur.

For LILRB3 epitope-mapping studies, LILRB3 domain-mutant-transfected HEK293F cells were stained with the relevant LILRB3 mAb for 25 minutes at 4°C, washed twice, stained with an anti–human-PE secondary antibody (109-116-170 Jackson ImmunoResearch) for 20 minutes at 4°C, before washing and acquisition using a FACSCalibur.

For staining of 2B4 reporter cells expressing LILR-A1, -A2, -A5, -B1, -B2, -B3, -B4, or -B5 (or nontransfected controls) (30, 62), cells were stained with 10 μg/mL LILRB mAb and incubated at 37°C with 5% CO_2_, overnight. The following day, the cells were washed and stained with a secondary anti-hIgG antibody (Jackson ImmunoResearch) at 4°C, for 45 minutes. The cells were washed and acquisition was performed using a FACScan (BD Biosciences), with data analyzed with FlowJo software.

### Surface plasmon resonance

SPR was performed with the Biacore T100 (GE Healthcare) as per the manufacturer’s instructions. LILRB3-hFc recombinant protein (extracellular LILRB3 domain with an hFc tag) was used as the ligand and immobilized by amine coupling onto a series S sensor chip (CM5). Various LILRB3 mAbs were used as analytes and flowed across the chip, and SPR was measured. *K_D_* values were calculated from the Univalent model of 1:1 binding by *K_d_*[1/s]/*Ka*[1/Ms], using the Biacore T100 Evaluation Software (GE Healthcare).

### T cell proliferation assay

PBMCs (1 × 10^7^ to 2 × 10^7^) were labeled with 2 μM CFSE at room temperature for 10 minutes. An equal volume of FCS was then added to quench labeling for 1 minute, before washing. Cells were subsequently resuspended in serum-free CTL medium (Immunospot) and plated at 1 × 10^5^ cells/well in a 96-well round-bottom plate (Corning). Cells were then stimulated with 0.02 μg/mL CD3 (clone OKT3, in-house), 5 μg/mL CD28 (clone CD28.2; BioLegend), and 10 μg/mL LILRB3 antibodies or a relevant isotype. Plates were then incubated at 37°C for 4 days, after which time cells were stained with 5 μg/mL CD8-APC (clone SK1; BioLegend), harvested and CFSE dilution measured by flow cytometry, as a readout for T cell proliferation.

### HSPC isolation and generation of humanized mice

Humanized mice were generated, as described (36). In brief, human fetal livers were obtained from aborted fetuses at 15–23 weeks of gestation, in accordance with the institutional ethical guidelines (Advanced Bioscience Resources, Inc.). All women gave written informed consent for the donation of their fetal tissue for research. Fresh tissue was initially cut into small pieces and digested with collagenase VI (2 mg/mL; Roche) for 30 minutes at 37°C. Single-cell suspensions were prepared by passing the digested tissue through a 100 μm cell strainer (BD Biosciences). HSPCs were purified using a CD34^+^ selection kit (STEMCELL Technologies); the purity of CD34^+^ cells was 90%–99%, as verified by CD34-PE (clone 561, BioLegend) immunophenotyping. Viability was determined through trypan blue exclusion of dead cells. All cells were isolated under sterile conditions and injected into NOD/SCID IL-2Rγ^–/–^ (NSG) mice.

NSG mice were purchased from The Jackson Laboratory and maintained under specific pathogen–free conditions in the animal facilities at Massachusetts Institute of Technology (MIT). To reconstitute mice, newborn pups (less than 2 days old) were irradiated with 100 cGy using a gamma radiation source and injected intracardially with CD34^+^ cells (~2 × 10^5^ cells/recipient), as reported previously (36). Around 12 weeks later, human leukocyte cell reconstitution of PBMCs was determined by flow cytometry and calculated as follows: % human CD45^+^ cells/(% human CD45^+^ cells + % mouse CD45.1^+^ cells). Mice with at least 40% human CD45^+^ leukocytes were used in subsequent experiments.

### In vivo allograft assay

Fully reconstituted humanized mice were injected with 200 μg LILRB3 mAb (clone A1) or an isotype-matched (hIgG1) control on day 0 and day 4, i.v. and i.p., respectively. On day 7, cohorts of mice were injected i.p. with 1 × 10^7^ luciferase-positive human “double-hit” B cell lymphoma cells (36, 37), derived from unrelated donors. Lymphoma cell growth was monitored over time using an IVIS Spectrum bioluminescent imaging system, as before (36). Mice with palpable tumors were sacrificed and Kaplan-Meier survival curves plotted.

### Transcriptome analysis of treated monocytes

To assess LILRB3-mediated transcriptional changes on monocytes, human PBMCs were isolated from freshly prepared PBMCs taken from healthy donors using an EasySep Human Monocyte Enrichment Kit (negative selection cell; STEMCELL Technologies). Cells were incubated in CTL medium (Cellular Technology Limited) supplemented with 100 U/mL penicillin-streptomycin, 2 mM glutamine, and HEPES buffer and treated with 10 μg/mL of an isotype control or an agonistic LILRB3 mAb (clone A1; hIgG1). Eighteen hours later, cells were lysed in RLT lysis buffer (QIAGEN) containing β-mercaptoethanol, and total RNA was extracted using the RNeasy Micro Kit (QIAGEN). Total RNA was assessed for quality and quantified using a total RNA 6000 Nano LabChip on a 2100 Bioanalyzer (Agilent Inc.), and cDNA libraries were prepared and sequenced according to the Illumina TruSeq RNA Sample Preparation Guide for SMARTer Universal Low Input RNA Kit (Clontech) and a HiSeq 2000 system (Illumina). RNA-Seq outputs were aligned to hg19 using Bowtie2 v2.2.3 (63). The number of mapped reads was quantified by RSEM v1.2.15 (64). Differential expression analysis between paired samples before and after treatment was performed using edgeR (65) with *P* < 0.05 and >2 fold change cutoffs. Differentially expressed genes were annotated using the online functional enrichment analysis tool Database for Annotation, Visualization and Integrated Discovery (http://david.ncifcrf.gov/) (66). GSEA was performed using Broad Institute Software (67), with the gene list preranked according to log fold change values from the edgeR output. For comparison of gene set expression, M1 and M2 macrophage gene sets (50) were obtained from the Molecular Signature Database (http://software.broadinstitute.org/gsea/msigdb/). Heatmaps were visualized with MeV (68). Raw sequences have been deposited in the National Center for Biotechnology Information’s Gene Expression Omnibus with accession ID GSE151675.

### Quantification of monocyte length

Human PBMCs were isolated as indicated above and then cultured for approximately 18 hours in 6-well plates in the presence of either isotype control or LILRB3 mAb (clone A1). Images were captured on an Olympus CKX41 inverted microscope, running under Olympus cellSens Standard software (version 2.1), and analyzed using ImageJ software (NIH). Length (μm) of monocytes was measured using the fragmented line tool, with the criterion of a maximum of 5 fragmented lines and exclusion of dead cells and cells found on the edges of the images. A total of 200–500 individual cell lengths were quantified from 3 independent donors.

### qPCR of treated monocytes

Probe-based qPCR was used to amplify cDNA in 20 μL reactions performed in triplicate for each sample condition in a PCR plate (Bio-Rad), as per the manufacturer’s protocol. The 96-well plate was run on a C1000 Thermal Cycler CFX96 Real-Time PCR System machine (Bio-Rad). CFX manager software (Bio-Rad) was used for data acquisition and analysis of gene expression initially recorded as cycle threshold values (Ct). The Ct values were normalized to housekeeping gene GAPDH and standardized to gene expression levels in isotype control–treated samples.

### Statistics

One-way ANOVA was performed for both the human leukocyte immunophenotyping and T cell proliferation data; straight bars indicate median values. On bar graphs, where at least 3 experiments were performed, error bars represent standard deviation. Kaplan-Meier plots were analyzed by log-rank test. One-way ANOVA with Bonferroni’s multiple-comparisons test were performed for qPCR data analysis. Statistical analysis was performed using GraphPad Prism (v6-8). *P* < 0.05 was considered significant.

### Study approval

All research with human samples and mice was performed in compliance with institutional guidelines, the Declaration of Helsinki, and the US Department of Health and Human Services *Guide for the Care and Use of Laboratory Animals* (National Academies Press, 2011). The Committee on Animal Care at MIT reviewed and approved the studies described here. All human samples (adult peripheral blood and fetal liver) were collected anonymously with informed consent by a third party and purchased for research. For human peripheral blood, ethical approval for the use of clinical samples was obtained by the Southampton University Hospitals NHS Trust, from the Southampton and South West Hampshire Research Ethics Committee following provision of informed consent.

## Author contributions

AR and MSC were grant holders, initiated the research proposals, supervised the project, and wrote the manuscript. MY, TS, UM, UCT, BH, AL, and MM generated human LILRB3 mAbs and performed the initial screening. MY characterized the mAbs and performed the in vitro functional assays. HTCC supported the molecular biology and CP and JSM supported the in vitro assays. JT provided reagents and edited the manuscript. BF provided expert advice on the mAb generation and edited the manuscript. JC provided expert advice on the in vivo assays and edited the manuscript. AR performed the in vivo experiments in humanized mice. DCJ designed DNA constructs used to generate antibodies, performed specificity assays against LILR transfectants, and edited the manuscript. CIM performed SPR assays. GH and SMT analyzed the RNA-Seq data. IT and MJG provided expert advice on the epitope mapping and functional assays, respectively, and edited the manuscript.

## Supplementary Material

Supplemental data

## Figures and Tables

**Table 1:**
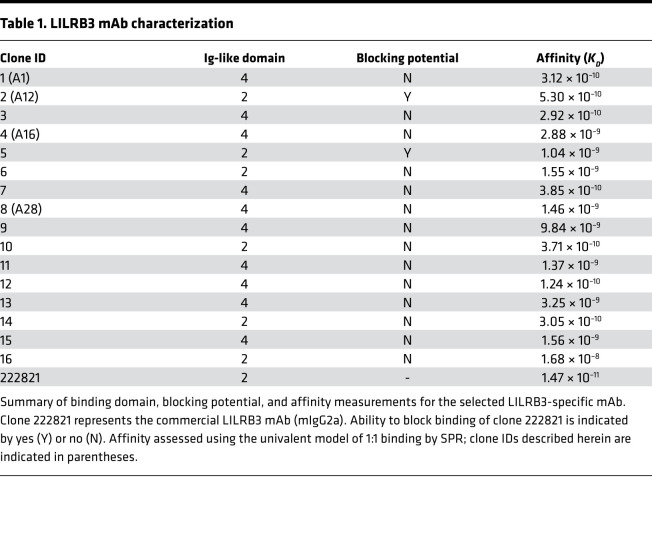
LILRB3 mAb characterization

## Version 1 09/01/2020

In-Press Preview

## Version 2 09/17/2020

Electronic publication
