# Supplementary material for: LILRB3 (ILT5) is a myeloid cell checkpoint that elicits profound immunomodulation
Source: JCI Insight. 2020 Sep 1;5(18):e141593. doi: 10.1172/jci.insight.141593 (PMC7526549; doi:10.1172/jci.insight.141593)
Supplement: Supplemental data [file jciinsight-5-141593-s062.pdf]

Supplemental Figures

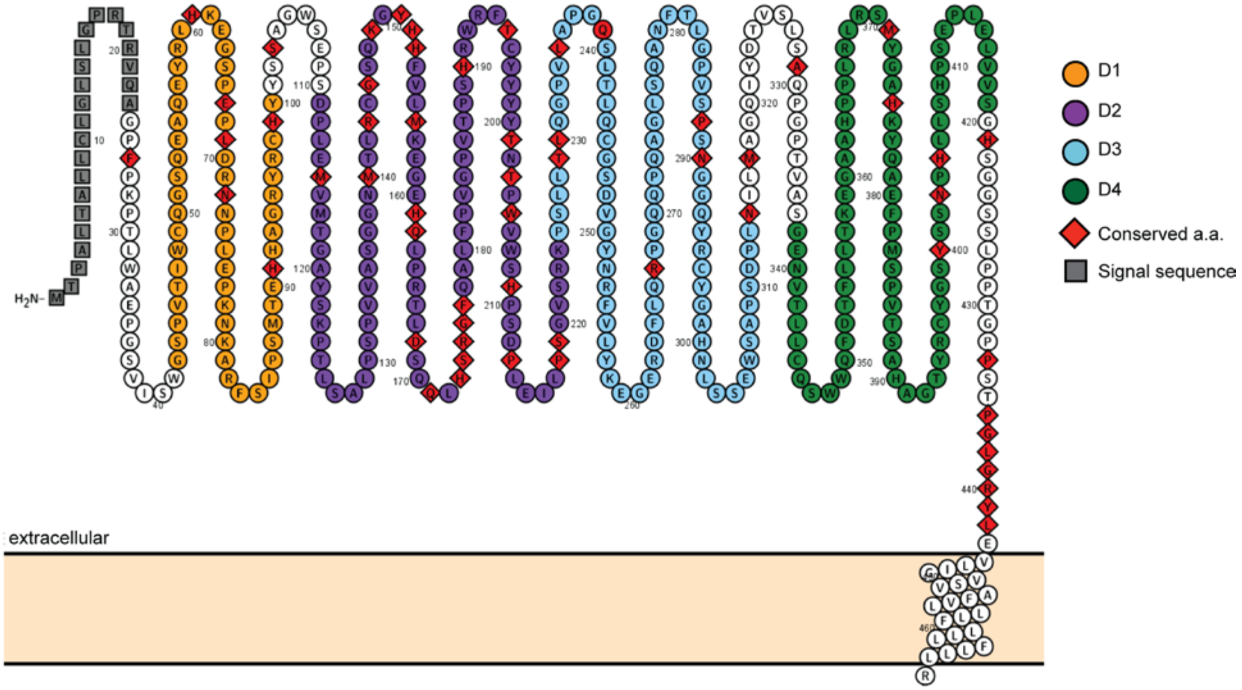

Supplemental Figure 1. Topological structure of LILRB3 ectodomain. Topological representation of the predicted extracellular domains of human LILRB3 (accession no. O75022) and its conserved a.a. residues, as aligned against LILRB1 (accession no. Q8NHL6), LILRB2 (accession no. Q8N423), LILRB4 (accession no. Q8NHJ6) and LILRB5 (accession no. O75023). Each predicted Ig-like domain (D1-4) has been separately color-coded and the conserved a.a. residues identified by red rhombus symbols. Source: UniProt and Protter.

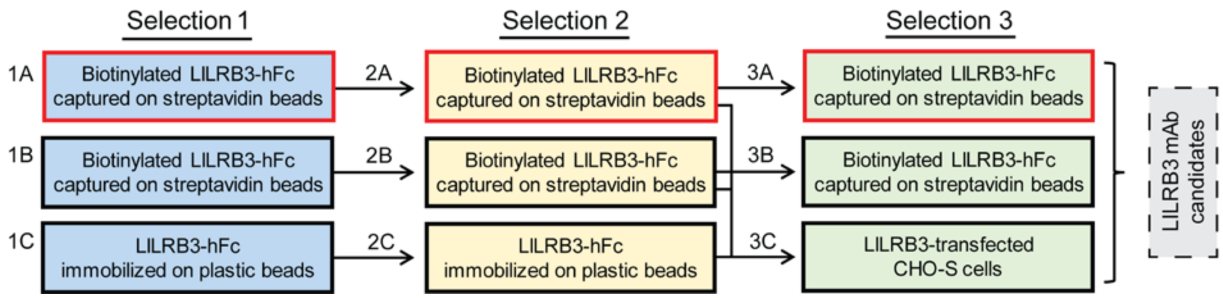

Supplemental Figure 2. Schematic of LILRB3-specific mAb selection strategy. Three ‘selection’ rounds were performed to generate LILRB3-specific mAbs by phage display. Three different methods to display the target protein were utilized: biotinylated target captured on streptavidin magnetic beads with competition *(A)*, biotinylated target without competition *(B)*, and immobilized protein coated on plastic polystyrene beads in the first two selection rounds, followed by cells expressing the target in selection around three using all phages from all three techniques used in panning two *(C)*. Throughout the panning rounds, LILRB1 was used as a non-target and as a competitor for LILRB3. 50 nM biotinylated non-target was used in the pre-selection for strategies where biotinylated protein was used *(A, B)*. 10 µg/ml non-target was used for strategies where coated protein was used *(C)*. No pre-selection was required for the cell strategies *(3C)*. Red border indicates competition with excess non-target protein was performed.

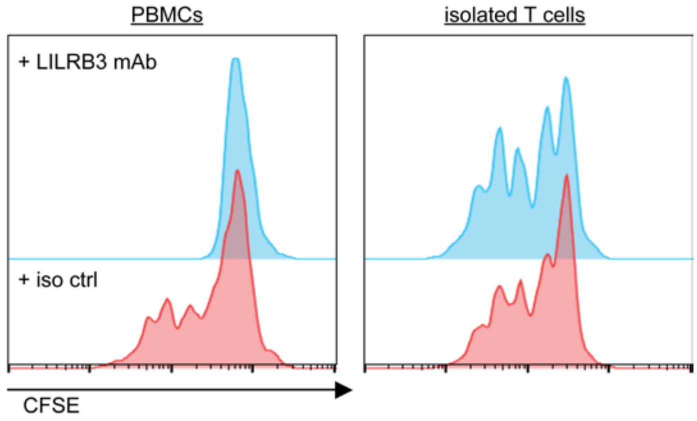

Supplemental Figure 3. T cell proliferation is not affected by agonistic LILRB3 mAb in the absence of myeloid cells. Fresh PBMCs or purified T cells, isolated from matched PBMCs using a human CD3 negative selection kit, were CFSE-labeled and then cultured in the presence of soluble 0.02 µg/ml or 5 µg/ml plate-bound OKT3 mAb and either 10 µg/ml of isotype control (pink) or an agonistic LILRB3 mAb (blue). Proliferation was measured through CFSE dilution on day 4 by flow cytometry. Representative histogram of 3 independent experiments shown for each culture.

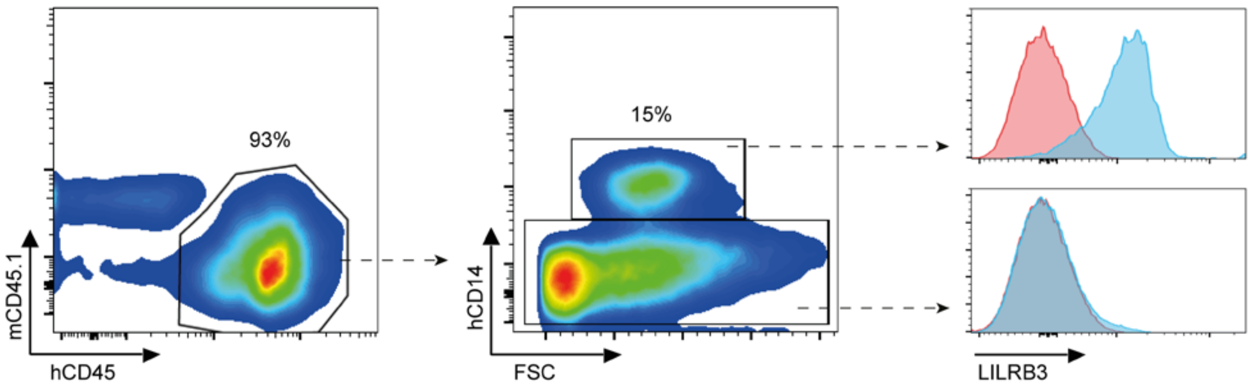

Supplemental Figure 4. Expression of LILRB3 on bone marrow-resident myeloid cells in humanized mice. Expression of LILRB3 on freshly isolated hCD45$^+$ bone marrow myeloid cells in humanized mice. Representative (3 mice/group, from 3 independent HSPC donors) flow cytometry plots (gated on live single cells) showing gating strategy and the restricted expression of LILRB3 on hCD14$^+$ myeloid cells ~3 months post engraftment of HSPCs; isotype control in pink, LILRB3 in blue.
